# QbD green analytical procedure for the quantification of tolvaptan by utilizing stability indicating UHPLC method

**DOI:** 10.1186/s13065-024-01214-2

**Published:** 2024-06-28

**Authors:** Shadab Anwar Hashmi, Pallavi Alegete

**Affiliations:** 1 Department of Analytical and Structural Chemistry, CSIR-Indian Institute of Chemical Technology, Hyderabad, Telengana 500007 India; 2 Academy of Scientific and Innovative Research (AcSIR), Ghaziabad, 201002 Uttar Pradesh India

**Keywords:** Tolvaptan, Quality by Design, Design Expert, Green Analytical Tools

## Abstract

For the first time a new QbD-assisted green stability indicating ultra-high-performance liquid chromatography (UHPLC) method was developed and validated for quantifying Tolvaptan. The method is simple, quick, cost-effective, and stable, and it was used to formulate a quality target product profile (QTPP) with strategically defined critical analytical attributes (CAAs) to meet specific criteria. Chromatographic separation was undertaken using a 10 cm long column of ACE excel super C18 with an interior diameter of 2.1 mm and particle size of 1.7 µm. The analysis was performed under controlled conditions at 25 ℃ with the mobile phase flowing at a rate of 0.2 mL/min and detection occurring at 220 nm. Injected 3 µL of standard by using an isocratic mobile phase system consisting of acetonitrile and water in a 95:5 v/v ratio. The diluents, prepared by mixing acetonitrile with water at a 90:10 volumetric ratio, were utilized. The analyte’s retention time was determined to be 1.63 min. The developed method provided reliable results with accuracy exceeding 99% and a correlation coefficient exceeding 0.999 ranged between 10 and 150 µg/mL across the range for LOQ—150% levels. Notably, during forced degradation testing, Tolvaptan exhibited susceptibility to acidic hydrolysis. The method effectively separated degradation products during stress testing, demonstrating its stability-indicating status. Environmental sustainability assessment of the developed method was conducted through the investigation of various indicators of Complex GAPI, Analytical Eco scale and Analytical GREEness and it was concluded the optimized method aligns with environmentally friendly practices.

## Introduction

Tolvaptan is a non-peptide antagonist that selectively inhibits the arginine vasopressin 2 receptor. It was synthesized by Otsuka Pharmaceutical Co., Limited, based in Tokyo, Japan [1] Tolvaptan received approval in Japan for the management of excessive fluid accumulation in patients with cirrhosis and heart failure when usual diuretics like loop diuretics, and thiazides become ineffective [2]. In 2009, Tolvaptan received approval from the Food and Drug Administration (FDA) for the management of clinically significant euvolemic and hypervolemic hyponatremia, particularly in cases where serum sodium levels are below 125 mEq/L. Tolvaptan is prescribed for hyponatremia associated with cirrhosis, congestive heart failure, and the inappropriate antidiuretic hormone syndrome (SIADH). Additionally, it has been authorized for the treatment of autosomal dominant polycystic kidney disease [3] and hepatic edema [4]. Traditional approaches to managing these patients often result in worsening hyponatremia and other electrolyte imbalances by promoting the loss of both water and electrolytes. However, the use of a specific vasopressin V2 receptor antagonist has the potential to counteract the effects of elevated AVP levels, leading to a reduction in water retention without disrupting the balance of electrolytes [5].

**Fig. 1:**
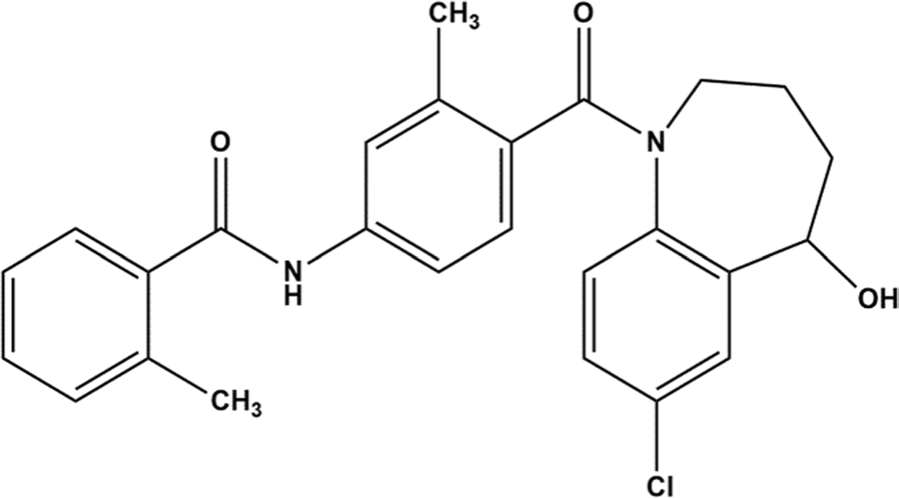
Structure of Tolvaptan

Tolvaptan is a racemic mixture of (±)-4’-[(7 chloro-2,3,4,5-tetrahydro-5-hydroxy-1H-1-benzazepin-1-yl) carbonyl]-o-tolu-m-toluidide, and is a crystalline powder that appears milky, with a molecular weight of 448.94 g/mol. Its empirical formula is C_26_H_25_ClN_2_O_3_. Tolvaptan is classified as a BCS Class IV medication due to its poor solubility (~ 0.1 mg/250 mL) [6]. It demonstrates solubility in ethanol and methanol, as well as high solubility in benzyl alcohol, while remaining insoluble in water. Tolvaptan undergoes primary metabolism in the liver through the action of CYP3A4 and is later excreted in the feces [7]. The structure of the Tolvaptan is illustrated in Fig. 1.

In light of the growing imperative for greener analytical techniques, it is crucial to consider and justify the key aspects when proposing or selecting an analytical method for determining a specific analyte [8]. Green analytical chemistry is gaining prominence and its adoption of environmentally friendly substances in diverse analytical approaches, especially in liquid chromatography, has become increasingly essential. Green analytical chemistry does not revolve solely around the surveillance of environmental pollutants,instead, it focuses on the transformation and environmentally friendly improvement of methodologies [9].

The evaluation of the environmental repercussions of chemical processes poses a significant challenge within the realm of green chemistry. Standardized measurement procedures empower us to compare the environmental impact of established solutions against newly developed alternatives, making it easier to recognize and opt for the most environmentally friendly choices. Several green metric tools, such as Green Analytical Procedure Index (GAPI), Complex GAPI, Analytical Eco-Scale (AES), National Environmental Methods Index (NEMI), Analytical GREEnness (AGREE), Analytical Method GREEnness Score (AMGS), AGREEprep, Analytical Method Volume Intensity (AMVI), Modified NEMI, HPLC-EAT (Environmental Assessment Tool), and other metric tools [10] have been created and are used to evaluate the environmental impact of analytical procedures.

The analysis of Tolvaptan using various HPLC techniques have been widely reported in literature that has been summarized in below Table 1. The reported methods were not suitable for high-throughput and cost-effective quality control analysis due to their lengthy runtime, expensive organic modifiers, and high flow rate. However, there is a lack of research on environmentally friendly HPLC methods that demonstrate stability with the QbD approach for the estimation of Tolvaptan, as guided by ICH guidelines. To the best of our knowledge, this is the first ever QbD assisted green analytical procedure for the quantification of Tolvaptan by utilizing stability indicating UHPLC method**.**

**Table 1:** Comparison of reported analytical methods for the estimation of Tolvaptan

S. No.	Titles	Chromatographic conditions	Retention time	Observation	References
1	Method Development and Validation of RP-HPLC Method for the Estimation of Tolvaptan in Bulk and its Tablet Dosage Form	Column: symmetry C18 (150 4.6 mm; 5 μm) Mobile phase: Acetonitrile: methanol: buffer (680 mg potassium dihydrogen phosphate in 500 ml water, pH-3adjusted with ortho-phosphoric acid) in the ratio of 40:10:50 v/v flow rate: 1.5 mL/min. Temp: room temp Wavelength: 260 nm	7.419 min	Method validation was performed but force degradation study was not done Phosporic acid buffer	Boggula et al. [26]
2	Development and Validation of RP-HPLC Method For Estimation of Tolvaptan in Bulk and its Pharmaceutical Formulation	Column: AMCHEMTEQ-USA-ACI C18 (150 mm × 4.6 mm; 5 µm) Mobile phase: Water: Acetonitrile (40:60) Flow rate: 1 mL/min Temp: 25 °C Wavelength: 254 nm	5.224 min	Development and validation were done through HPLC and there was no forced degradation data performed Forced degradation studies were not done	Chakravarthy and Shankar [27]
3	Method Development and Validation of Tolvaptan in its API and Formulation by using PDA Detector-RP-HPLC	Column: Nucleosil-C18 (250 × 4.6 mm; 5 µm) Mobile phase: Buffer of Sodium dihydrogen phosphate: Acetonitrile (45:55%V/V) or 10:90 v/v Flow rate: 1 mL/min Temp: ambient temp Wavelength: 264 nm	4.536 min	Conventional method with no forced degradation data Phosphate related buffer	Anusha and Kalaichelvi [28]
4	Analytical Method Development and Validation of Tolvaptan and its Related Substances in Drug Product by RP-HPLC	Column: Inertsil ODS-3V (250 × 4.6 mm; 5 μm) Mobile phase: Mobile phase: A: 0.1% H_3_PO_4_ Solution in 1000 mL H_2_0 Mobile phase: B: Methanol: ACN (50:50) Flow rate: 1 mL/min Temp: 35 °C Wavelength: 254 nm	13.96 min	Tolvaptan and its Related impurities were done in drug product and performed method validation Phosporic acid buffer	Chandmalin and Rao [29]
5	An Eco-friendly RP-HPLC and UV-Method Development and Validation for an Estimation of Tolvaptan in Bulk and Tablet Dosage form Followed by Forced Degradation Studies	Column: Sunsil C18 column (150 mm × 4.6 mm; 5 µm) Mobile phase: Acetonitrile: Water [45:55] Flow rate: 1 mL/min Temp: ambient temp Wavelength: 266 nm	4.7 min	HPLC method with validation and stress studies were performed and reported Run time is more and the method is not driven by QbD	Patel et al. [30]
6	A New Approach for Analytical Method Development and Validation for Quantification of Tolvaptan Using RP-HPLC in Bulk and in its Tablet	Column: WatersC18 100 mm × 4.6 mm, 5 µm Mobile phase: Methanol: Water (Upgrade)adjusted to pH-3 with GAA (60:40) Flow rate: 0.7 mL/min Temp: ambient temp Wavelength: 267 nm	3.4 min	Degradation studies were not conducted Glacial Acetic acid was used for pH adjustment Run time was more and the method was not driven by QbD	Bhavyasri et al. [31]
7	Enantioselective Analysis of Tolvaptan in Rat and Dog Sera by High Performance Liquid Chromatography and Application to Pharmacokinetic Study	Column: CHIRALCEL OD-R (250 mm × 4.6 mm, 5 µm) Mobile phase: Acetonitrile–water–acetic acid (55:45:1, v/v/v) Flow rate: 1 mL/min Temp: 40 °C Wavelength: 265 nm	8.8 min	Pharmacokinetic Study on rat and Dog and sera was performed Acetic acid was used in the mobile phase	Furukawa et al. [32]
8	A New Stability Indicating RP-HPLC Method Development and Validation for the Estimation of Tolvaptan with Forced Degradation Studies in Bulk and Tablet	Column: ODS 250 × 4.6 mm, 5 µm Mobile phase: 60:40 (v/v) mixture of acetonitrile and 0.1% orthophosphoric acid buffer Flow rate: 1.0 mL/min Temp: 30 °C Wavelength: 270 nm	2.59 min	Both method development and validation force degradation study were done in bulk and tablets Orthophosphoric acid buffer was used in the mobile phase	Ganipisetty et al. [33]
9	Development and Validation of Novel Stability Indicating RP- HPLC Method for Quantification of Tolvaptan in Bulk and Pharmaceutical Dosage Form	Column: ODS-3v column, 150 × 4.6mm, 5.0 mm Mobile phase: Orthophosphoric acid and acetonitrile as solvent in the ratio of 40:60 (v/v) Flow rate: 1 mL/min Temp: 30 °C Wavelength: 254 nm	2.59 min	Conventional method development and validation were performed in bulk and pharmaceutical dosage forms Orthophosphoric acid buffer was used in the mobile phase	Khaleela and Rahaman [34]
10	Rapid RP-HPLC Method Development and Validation of Tolvaptan in Bulk and Pharmaceutical Dosage Form for an Internal Standard	Column: Eclipse C18 column (100 mm × 4.6 mm, 3.5 μm particle size) Mobile phase: Methanol:0.2M phosphate buffer (70:30v/v) Flow rate: 1.0 mL/min Temp: 25 °C Wavelength: 267 nm	3.68 min	Internal standard was used with a conventional mobile phase and performed method development and validation orthophosphoric acid buffer was used in the mobile phase	Vijaya Sri et al. [35]
11	Application of Response Surface Methodology in Development and Optimization of Stability Indicating RP-HPLC Method for Determination of Tolvaptan in Bulk and Formulation	Column: Kromasil C18 (250 mm × 4.6 mm, 5 µm) Mobile phase: Acetonitrile and phosphate buffer with pH 5.5 (70:30% V/V) Flow rate: 1.0 mL/min Temp: 25 °C Wavelength:	3.24 min	Design of experiment was performed with conventional method in bulk and formulation Phosphate buffer was used Run time was more & method not driven by green analytical approaches	Sutar and Magdum [36]
12	A New Stability-Indicating and Validated RP-HPLC Method for the Estimation of Tolvaptan in Bulk and Pharmaceutical Dosage Forms	Column: C18 (100 mm × 4.6 mm I.D., 5 µm) Mobile phase: orthophosphoric acid and acetonitrile in the ratio of 35:65 V/V Flow rate: 1 mL/min Temp: 30 °C Wavelength: 254 nm	3.46 min	Routine traditional method in bulk and pharmaceutical dosage forms orthophosphoric acid buffer was used in the mobile phase	Bonthu et al. [37]
13	Quantification of Tolvaptan API by utilizing UHPLC method and stability indicating approach with validation by optimizing Design-Expert software	Column: ACE Excel super C18 100, 1.7 µm × 2.1 mm Mobile phase: acetonitrile and water in the ratio of 95:5 V/V Flow rate: 0.2 mL/min Temp: 25 °C Wavelength: 220 nm	1.63 min	A QbD-assisted green stability-indicating UHPLC method was optimized and achieved reduced runtime Forced degradation studies were performed, and method optimization was achieved by using the quality design based central composite design approach	Current method

A green analytical method was initiated on the AES, AGREE, AGREE prep and Complex GAPI tool for greenness assessment of the analytical procedure [11]. The proposed method is eco-friendly and adheres to the green analytical chemistry (GAC) principle. It is necessary to prioritize the development and implementation of such environmentally friendly techniques to minimize the adverse impact of chemical processes on the environment.

## Quality by design

The Quality by Design (QbD) [11] encourages proactive methodology to analytical quality, ensuring it's built into the process from the beginning, not just checked at the end. This proactive approach ensures that quality is ensured into the very design of the analytical procedure, leading to more reliable and consistent outcomes ICH Topic Q8 [12] Pharmaceutical Quality by Design (QbD) has undergone advancements with the introduction of ICH Q10 (Pharmaceutical Quality System), ICH Q9 (Quality Risk Management), and ICH Q8 (R2) (Pharmaceutical Development) throughout the years [13–15]. One notable benefit of employing the QbD technique lies in its adaptability to conduct a qualification aligned with the distinct Analytical Target Profile established for the method's intended utilization [16].

The QbD methodology offers a comprehensive framework for process optimization, enabling the identification of product features and the origins of inconsistencies [17]. It begins with setting the objective and then defining the Quality Target Product Profile (QTPP), encompassing essential requirements for safety, efficacy, and quality [12]. These prerequisites form the basis for product design and must be consistently replicated to achieve the aimed benefits. Recent developments in combining green analytical chemistry with the Quality-by-Design approach were discussed [18, 19].

Employing risk-assessment tools, further investigation into CPPs and CQAs is carried out through a Design of Experiment (DOE) study. DOE is a statistical technique that allows for the systematic investigation of systems and processes to comprehend the primary and interactive impacts of diverse CPPs. Additionally, it offers flexibility by forecasting the magnitude of these interactions [20].

The Design of Experiments (DoE) methodology involves the organized variation in controlled input factors to assess their impact on output responses. This systematic variation facilitates the identification of crucial input factors, the determination of optimal settings for these factors resulting in optimized output responses, and the elucidation of interactions among the input factors. The choice of an optimal experimental design should take into account various factors, including well-defined objectives, the number of input factors and interactions under examination, statistical validity and efficiency inherent in each design. In this paper we used Design-Expert software for doing the DoE and further analysis was conducted to assess robust chromatographic conditions.

To the best of our knowledge for the first time we have developed and validated a QbD assisted stability indicating green UHPLC method that leads to the development of more resilient methods, generating consistent, reliable, and high-quality data. Consequently, it reduces the likelihood of regular incidents in routine environments, saving both time and resources by minimizing the need for investigations.

## Materials and methods

### Chemicals and standards

Tolvaptan (API) with a purity of 99% was attained as a gift sample from Extrovis, located in Hyderabad, Telangana, India. Consistently throughout the study, we employed HPLC-grade acetonitrile procured from E. Merck (India) Ltd. in Mumbai. To achieve HPLC-grade water, we utilized a Milli-Q Plus water purification system sourced from Millipore in Milford, MA, USA.

### Instrumentation

The method development and validation were done using an Agilent UHPLC system (Infinity 1220 LC) coupled with a Diode Array Detector (DAD). Information gathering and subsequent analysis were conducted with Open LAB software and ACE Excel super C18 (100 mm × 2.1 mm, 1.7 μm) column was utilized for analysis.

### Analytical solutions

#### Preparation of mobile phase

A combination of acetonitrile and water, stored in two separate 1-L bottles. The ratio of acetonitrile to waterwas 95:5% of the total flow.

#### Diluents

Diluents were made by mixing acetonitrile and water with a volumetric ratio of 90:10.

#### Stock solution preparation

About precisely weighing, 10 mg of Tolvaptan API was added to a 10 mL volumetric flask. The API was then dissolved in 100% acetonitrile to obtain a concentration of 1000 µg/mL.

#### Blank standard

Diluent was taken as blank.

#### Standard solution preparation.

Standard preparation of 100 µg/mL solution was made through taking 1 mL of the stock solution and 9 mL diluent was added to it. Different concentration of test solution was made from the stock solution by dilution calculation to get the desired concentration for specific concentration for method validation parameters.

### Operating chromatographic conditions

The isocratic mobile phase comprising Acetonitrile: Water [95:5 v/v], was passed through the column with a consistent flowing rate of 0.2 mL/min at room temperature of 25 °C with an injection volume of 3 µL for a total of 5 min. The stationary phase employed was the ACE Excel Super C18 column (100 mm × 2.1 mm) and 1.7 µm size of particles of silicones. The other experimental conditions before the operating chromatographic conditions were given in Table 2. The UV–visible detector remained set at a detection wavelength of 220 nm.

**Table 2:** Different trials for method development for Tolvaptan

S. No.	Chromatographic conditions	Observations	Reference figure
Different column			
1	Column- Phenomenex Luna C 18 250 × 4.6 mm (10 µm) M.P: Acetonitrile and water (95:5) Temp: 25 °C Flow: 1 mL/min Inj. Volume: 3 µL Time: 10 min Diluents: Acetonitrile and water as 90:10	Peak shape is adequate but a short Retention Time required	Figure 2
2	Column: Phenomenex Luna C18 250 × 4.60 mm (5 µm) M.P: Acetonitrile and water (95:5) Temp: 25 °C Flow: 1 mL/min Inj. Volume: 10 µL Time: 10 min Diluents: Acetonitrile and water as 90:10	Peak splitting (peak shape was not adequate)	
3	Column: Eclipse plus C18 100 × 4.60 mm (3.5 µm) M.P: Acetonitrile and water (95:5) Temp: 25 °C Flow: 0.5 mL/min Inj. Volume- 3 µL Time: 5 min Diluents: Acetonitrile and water as 90:10	Peak shape was not adequate, tailing factor > 2	
4	Column: ACE Excel C18 100 × 2.1 mm (2 µm) M.P: Acetonitrile and water (95:5) Temp: 25 °C Flow- 0.2 mL/min Inj. Volume: 3 µL Time: 5 min Diluents: Acetonitrile and water as 90:10	Peak shape not adequate, theoretical plate < 2000 main peak starts with another peak, no clear separation	
Different mobile phase with different flow rate			
5	M.P: Acetonitrile and Methanol in 95:5 Column: Ace Excel Super C18 Temp: 25 °C Flow: 0.1 mL/min Inj. Volume: 3 µL Time: 5 min Diluents: Acetonitrile and water as 90:10	Theoretical plates are too low (lot lesser than 2000)	Figure 3
6	M.P –Acetonitrile and Methanol in 95:5 Column- Ace Excel Super C18 Temp- 25 °C Flow-0.3 mL/min Inj. Volume- 3 µL Time- 5 min Diluents- Acetonitrile and water as 90:10	Retention Time is too high	
7	M.P: Acetonitrile and Methanol in 95:5 Column: Ace Excel Super C18 Temp: 25 °C Flow: 0.5 mL/min Inj. Volume: 3 µL Time: 5 min Diluents: Acetonitrile and water as 90:10	Theoretical plates are too low (less than 2000)	
8	M.P: 0.1%TFA in Acetonitrile and methanol in ratio 95:5 Column: Ace Excel Super C18 Temp: 25 °C Flow: 0.1 mL/min Inj. Volume: 3 µL Time: 5 min Diluents: Acetonitrile and water as 90:10	Peak not detected	Figure 4
9	M.P: 0.1%TFA in Acetonitrile and methanol in ratio 95:5 Column: Ace Excel Super C18 Temp: 25 °C Flow: 0.3 mL/min Inj. Volume: 3 µL Time: 5 min Diluents: Acetonitrile and water as 90:10	Theoretical plates are too low	
Different temperature and flow rate			
10	Column: ACE Excel Super C18 100 × 2.1 mm (2 µm) M.P: Acetonitrile and water (95:5) Temp: 45 °C Flow: 0.5 mL/min Inj. Volume- 3 µL Time: 5 min Diluents: Acetonitrile and water as 90:10	Theoretical plates and resolution are too low	Figure 5
11	Column: ACE Excel Super C18 100 × 2.1 mm (2µm) M.P: Acetonitrile and water ( 95:5) Temp: 45 °C Flow: 0.3 mL/min Inj. Volume: 3 µL Time- 5 min Diluents- Acetonitrile and water as 90:10	Theoretical plate and resolution are too low	
12	Column- ACE Excel Super C18 100 × 2.1 mm (2µm) M.P –Acetonitrile and water ( 95:5) Temp: 45 °C Flow: 0.2 mL/min Inj. Volume: 3 µL Time: 5 min Diluents: Acetonitrile and water as 90:10	Theoretical plates and resolution are good	
13	Column: ACE Excel Super C18 100 × 2.1 mm (2µm) M.P: Acetonitrile and water (95:5) Temp: 45 °C Flow: 0.1 mL/min Inj. Volume: 3 µL Time: 5 min Diluents: Acetonitrile and water as 90:10	Theoretical plates and resolution are good but RT is not adequate	
14	Column: ACE Excel Super C18 100 × 2.1 mm (2µm) M.P: Acetonitrile and water (95:5) Temp: 25 °C Flow: 0.5 mL/min Inj. Volume: 3 µL Time: 5 min Diluents: Acetonitrile and water as 90:10	Theoretical plates and resolution are too low	Figure 6
15	Column: ACE Excel Super C18 100 × 2.1 mm (2 µm) M.P:Acetonitrile and water (95:5) Temp: 25 °C Flow: 0.3 mL/min Inj. Volume: 3 µL Time: 5 min Diluents: Acetonitrile and water as 90:10	Theoretical plates are less than 2000 but resolution is good	
16	Column: ACE Excel Super C18 100 × 2.1 mm (2µm) M.P: Acetonitrile and water (95:5) Temp: 25 °C Flow: 0.2 mL/min Inj. Volume: 3 µL Time: 5 min Diluents: Acetonitrile and water as 90:10	Theoretical plates and resolution are good and can be adopted in the present method	
17	Column: ACE Excel Super C18 100 × 2.1 mm (2µm) M.P: Acetonitrile and water (95:5) Temp: 25 °C Flow: 0.1 mL/min Inj. Volume: 3 µL Time: 5 min Diluents: Acetonitrile and water as 90:10	Theoretical plates and resolution are good but RT is not adequate	

### Quality by design (QbD) execution

**Fig. 2:**
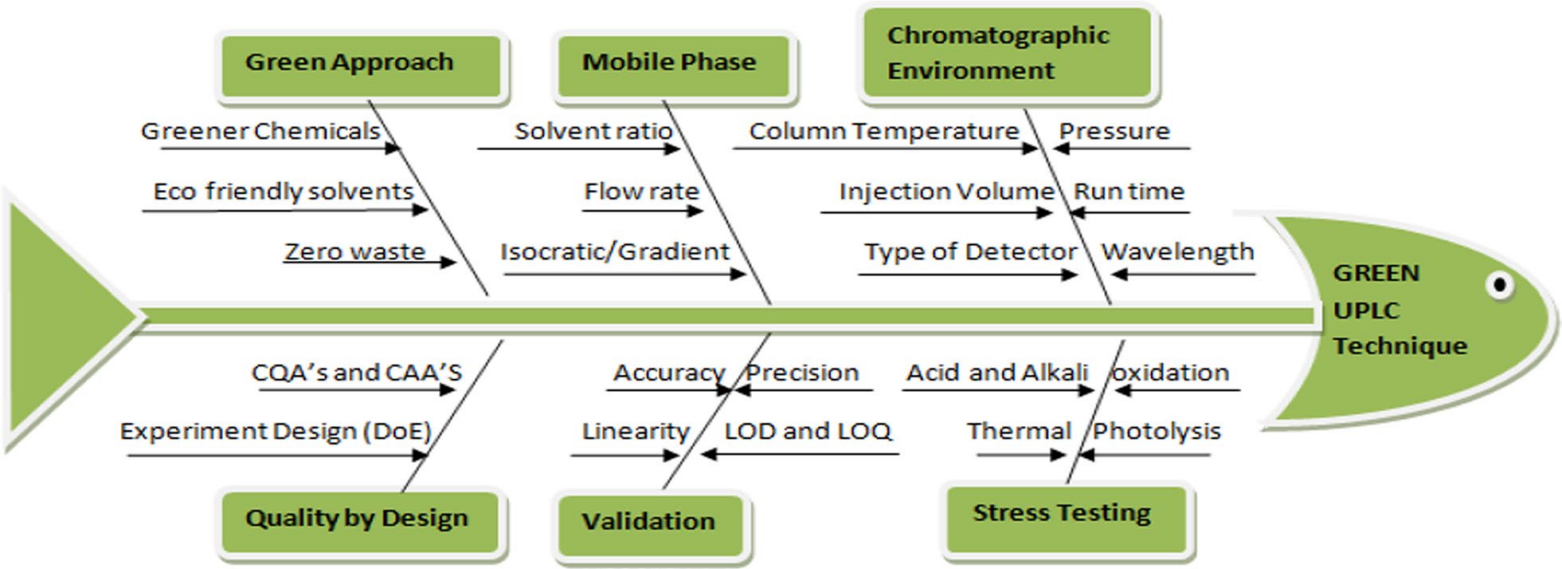
Fishbone diagram

The practice of Quality by Design (QbD) principles in the development of analytical techniques has gained widespread popularity as a well-established practice to achieve enhanced robustness and improved method performance. The implementation of Analytical Quality by Design (AQbD) methodology enables a comprehensive understanding of essential variability sources based on scientific principles and risk assessment. This involves identifying Critical Method Parameters (CMPs)/Critical Process Parameters (CPPs) (buffer pH and mobile phase ratio) through risk assessment and factor screening this CMPs further evaluation affecting the critical analytical attributes (CAAs)/CQA (peak response, peak tailing, theoretical plates number and retention time) Once CPPs and CQAs are identified, a risk assessment is performed to evaluate the potential implications of CPP variations on CQAs by DoE. Variable parameters are designed as a fishbone diagram in Fig. 2 which depicts all the factors that influenced on further research and investigation. The analysis of optimization data was conducted by stat ease Design-Expert software. Response surface factor (RSF) was used for experimental design it contains Central Composite Design (CCD), [11, 21]. used three factors around three centre points for 20 runs. The repetition of center points were done to ascertain repeatability, enhance the robustness of the experimental design, and assess pure error. The values were presented in Table 3

**Table 3:** Design summary layout

Std	Run	Factor 1	Factor 2	Factor 3	Response 1	Response 2	Response 3
		A:Flow rate	B:Temperature	C:Mobile phase composition	Retention time	Tailing Factor	Theoretical plates
		mL/min	C	mL	Min		
13	1	0.2	25	93	1.61	1.1	2298
18	2	0.2	25	95	1.63	1.2	2210
5	3	0.1	20	97	1.71	1.3	2273
3	4	0.1	30	93	1.71	1.2	2154
14	5	0.2	25	97	1.61	1.2	2269
9	6	0.1	25	95	1.71	1.3	2167
16	7	0.2	25	95	1.62	1.1	2202
15	8	0.2	25	95	1.61	1.2	2260
7	9	0.1	30	97	1.71	1.3	2156
4	10	0.3	30	93	1.51	1.1	2212
19	11	0.2	25	95	1.61	1.2	2243
1	12	0.1	20	93	1.7	1.1	2145
6	13	0.3	20	97	1.5	1.3	2234
11	14	0.2	20	95	1.61	1.2	2164
8	15	0.3	30	97	1.51	1.3	2251
17	16	0.2	25	95	1.63	1.2	2267
12	17	0.2	30	95	1.59	1.1	2121
2	18	0.3	20	93	1.54	1.1	2138
10	19	0.3	25	95	1.58	1.3	2278
20	20	0.2	25	95	1.63	1.2	2277

A comprehensive statistical analysis incorporating prediction equations, ANOVA, actual vs. predicated plots, lack-of-fit analysis, 3D plots were employed to thoroughly evaluate each (CQA)/CAA and define the experiment of design.

### Validation parameters

The established method underwent validation for linearity, accuracy, precision, specificity, robustness, limit of detection (LOD) and limit of quantification (LOQ), as per the “ICH Q2 (R1)”.

#### Linearity

Linearity in the validation parameter indicates the capability, within a specified range, to produce test results that are directly proportional to the concentration of the analyte in the sample.

Calibration solutions for the assay method were equipped at six concentrations between 10 µg/mL and 150 µg/mL. HPLC analysis confirmed linearity of the method, with peak areas plotted against drug concentrations and analyzed using least square regression.

#### Accuracy

Accuracy can be described as proximity of measured values to the actual or accepted values. This involves quantifying the quantity of the standard recovered involved conducting triplicate measurements at three distinct concentrations (50%, 100%, and 150%). The calculation includes determining the mean percentage recoveries at all levels, and the % RSD (relative standard deviation) is then computed.

#### Precision

Precision measures the degree of reproducibility of the analytical results. It indicates how consistently the method can produce the same results when repeated under the same conditions. Precision was determined at two levels: system precision and method precision. System precision involved five injections from the same standard solution. Assessment of method precision involved injecting six samples of the standard solution at a consistent concentration. The resulting peak responses were utilized to determine the average area, standard deviation and % RSD for both conditions.

#### Limit of detection (LOD) and limit of quantification (LOQ)

The lowest concentration of the analyte that can be accurately detected is known as LOD with a specified degree of confidence. It indicates the sensitivity of the method. The lowest amount of analyte that can be precisely and accurately quantified with a certain level of confidence is known as the limit of quantification. The LOD and LOQ were evaluated from the calibration curves by applying statistical calculations.

#### Robustness

Robustness defines the method's ability to withstand minor variations in conditions, such as column temperature, ratio of mobile phase, and flow rate. Flow rate were limited to ± 0.1 mL/min, mobile phase composition were considered as ± 1% v/v, and temperature changes were controlled with ± 5 °C. Duplicate injections of sample were executed and calculations were made for the average peak response, standard deviation and % RSD.

#### Solution stability

Solution stability pertains to the ability of the solutions to maintain their concentration, purity, and analyte integrity over a period of time under defined storage conditions. Standard solution was analyzed of day one and day seven to check the solution stability using the validated HPLC method and percentage change in peak area was calculated for above period of time.

#### Stability indicating stress study

The evaluation of the intrinsic stability of the drug molecule is facilitated through the utilization of  stress studies. Both FDA and ICH guidelines require the incorporation of stress studies to understand and examine alterations in the characteristics of both a drug substance and a drug product across different timeframes and under diverse environmental conditions [22, 23]. The ICH guidelines mandate the incorporation of stress studies involving altered conditions, encompassing oxidation, acidic, base pH, exposure to light, dry heat, and hydrolysis.

##### Oxidation

Hydrogen peroxide was used for oxidation of the solution. An electron transfer pathway is involved in the drug's oxidative breakdown. For oxidation 10 mg API was combined with 5 mL of acetonitrile (ACN), followed by the addition of 5 mL of 6% H_2_O_2_ solution, constituting the degradation solution and yielding a stock solution of 1000 µg/mL. This solution was refluxed for 6 h at 60 °C, Subsequently, a 100 µL aliquot of the solution was withdrawn and diluted to 1 mL with a dilution solution. This sample was then injected for HPLC analysis for to detect degraded products and separate them from the original peak.

##### Acidic hydrolysis

Acidic hydrolysis involves catalyzing the molecule's ionizable functional groups. Acid degradation, induced by either HCl or H_2_SO_4_ at varying concentrations ranging from 0.1 N to 5 N, is a common method. In this experiment, 0.2 N HCl was employed as the degradation solution. For acidic hydrolysis, 10 mg of Active Pharmaceutical Ingredient (API) was combined with 5 mL of acetonitrile (ACN), followed by the addition of 5 mL of 0.2 N HCl, resulting in a stock solution of 1000 µg/mL. The mixture underwent reflux for 6 hours at 60 °C. Subsequently, a 100 µL aliquot of the solution was withdrawn and diluted to 1 mL with a dilution solution. This sample was then injected for High-Performance Liquid Chromatography (HPLC) analysis to detect degraded products and separate them from the original peak.

##### Basic hydrolysis

The basic hydrolysis entails the catalytic breakdown of ionizable functional groups within the molecule. Alkali degradation induced by NaOH of different concentration from 0.1 N to 5 N. Here 0.2 N HCl was used as a degradation solution. Basic hydrolysis was conducted as follows: 10 mg of API was combined with 5 mL of acetonitrile (ACN), followed by the addition of 5 mL of 0.2 N NaOH, resulting in a stock solution of 1000 µg/mL. The solution was then refluxed for 24 hours at 60°C. Afterward, a 100 µL aliquot of the solution was withdrawn and diluted to 1 mL with a dilution solution. Subsequently, this sample was injected for High-Performance Liquid Chromatography (HPLC) analysis to detect degraded products and separate them from the original peak.

##### Thermal degradation

Sample of solid drug was exposed to dry heat for accelerated degradation, here 10 mg of API was kept in hot air oven for 5 days. A 100 µg/mL solution was prepared using a 1000 µg/mL stock sample and this resulting solution was utilized for the subsequent HPLC analysis.

##### Photolysis

Photo stability studies aim to produce primary degradants of the drug substance by subjecting it to UV or fluorescent conditions. The ICH guidelines specify conditions for photo stability testing [24] recommending exposure to light at least 1.2 million lux hours and 200 W hours per square meter for drug substance and solid/liquid drug product samples. The commonly acknowledged wavelength range for initiating photolytic degradation is 300–800 nm. Photo oxidation, induced by light stress conditions through a free radical mechanism, was also considered [25]. 10 mg of API was exposed to direct sun light for 3 days. Subsequently, a 1000 µg/mL stock solution was prepared from it, and a 100 µg/mL sample was drawn for analysis.

## Results and discussion

### Method optimization

The objective of this study was to develop a QbD assisted quick and environmentally friendly stability-indicating UHPLC method for the estimation of Tolvaptan. From the analytical QbD approach, CMPs have cause-and-effect relationship possessing the capacity to impact the chosen CAAs. The most vital CMPs are mobile phase composition, Diluents, flow rate, column oven temperature and length of the column; three pivotal CAAs are peak tailing, retention time, and theoretical plates. The Design of Experiments (DoE) methodology utilized a pinpoint central process and established a design space rooted in statistical significance. Validation of the method ensured a reliable confirmation of quality results. In the current study, CCD was created from Design-Expert software, using three factors such as flow rate, column temperature and acetonitrile composition in the mobile phase. A total of 20 experiments of face centered CCD were created with 8 factorial, 6 axial, and 6 center points [21]. The chosen factors, responses and experimental values are tabulated in Table 3. Subsequently, the experimental results from this data underwent statistical evaluation using the Design-Expert software. The CAAs of each response were analyzed using various statistical techniques such as prediction equations, ANOVA, actual vs. predicted plot, lack of fit, contour plot. Understanding the performance of the technique across various experimental scenarios offered a fundamental grasp of the method control approach. The involvement of the test quadratic polynomial models was assessed using ANOVA, and the corresponding results were showcased in Table 4.

**Table 4:** ANOVA fit statistics of proposed method

	Retention time	Tailing factor	Theoretical plates
Std. Dev	0.0158	0.0430	35.97
Mean	1.62	1.20	2215.95
C.V. %	0.9788	3.59	1.62
R^2^	0.9712	0.8456	0.7848
Adjusted R^2^	0.9452	0.7067	0.5911
Predicted R^2^	0.7561	0.0946	− 0.2803
Adeq. precision	20.1901	8.4016	6.8243
Lack of fit (p-values)	0.0713	0.4154	0.2872

**Fig. 3:**
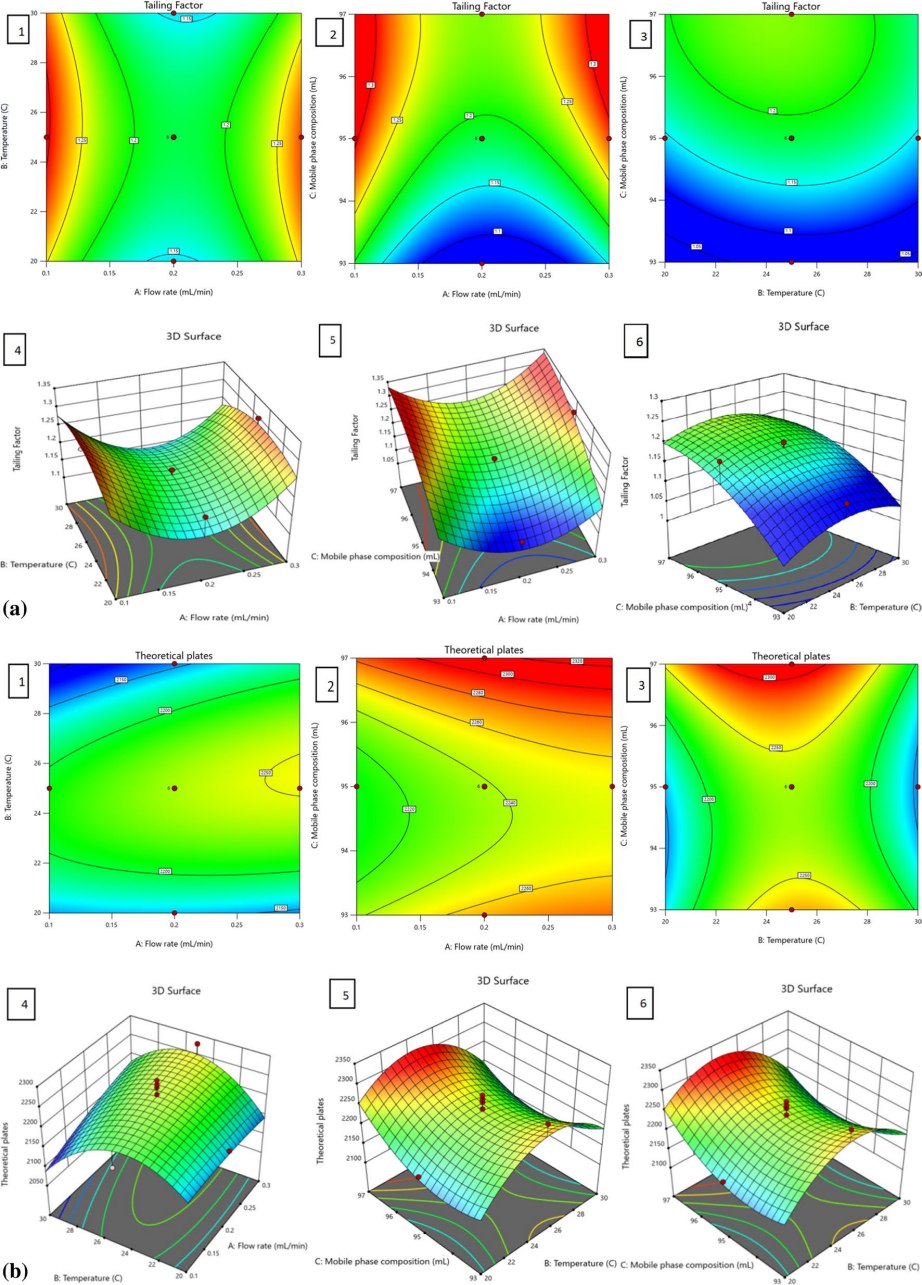

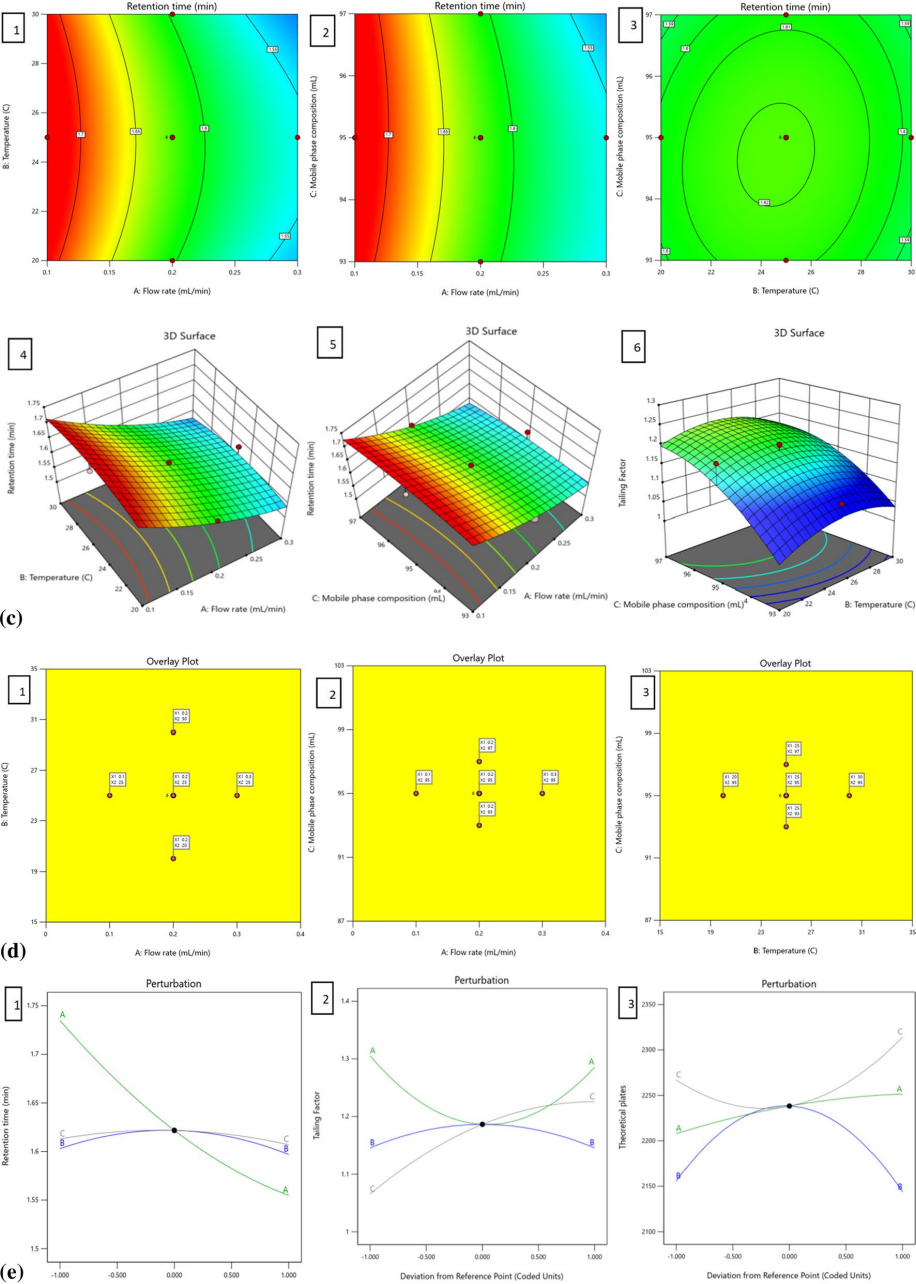
2D-contours (1-2-3) and 3D-response surface(4-5-6) plots showing the influence of CMPs, i.e., flow rate (**A**), temperature (**B**) mobile phase concentration (**C**) on retention time as the CAA. **b** 2D-contours (1–2-3) and 3D-response surface (4-5-6) plots showing the influence of CMPs, i.e., flow rate (**A**), temperature (**B**) mobile phase concentration (**C**) on tailing factor as the CAA. **c** 2D-contours(1–2-3) and 3D-response surface(4-5-6) plots showing the influence of CMPs, i.e., flow rate (**A**), temperature (**B**) mobile phase concentration (**C**) on theoretical plate as the CAA. **e** perturbation plot showing deviation from reference point with 1. Retention time 2. Tailing factor 3. Theoretical plate

The adjusted (Adj. R^2^) and predicted (Pred. R^2^) were utilized to assess the polynomial regression equations. The R^2^ values for the determination coefficients approached 1, indicating that the data closely adhered to the regression statistics line with an accuracy exceeding 99%. Extension studies were conducted to study the 3D response plots with 2D contour. The impact of factors such as the mobile phase composition, temperature and flow rate of the LC system on responses like retention time, theoretical plates, and tailing factor was unveiled through 3D response surface plots. Figure 3a–e displays representative graphs of these responses, demonstrating the intricate interaction between factors A and B and their interdependence, while factor C remained constant. The 3D surface plots illustrated a decrease in retention time with increasing flow rate, while changes in mobile phase composition and column temperature had negligible effects on retention time. The tailing factor was significantly influenced by flow rate and mobile phase composition, with the maximum tailing factor observed at low and high flow rates. However, the maximum tailing factor was observed under center point conditions of column temperature (25°C) and mobile phase composition (95%). The column theoretical plates were minimally affected by flow rate, with maximum values observed at the center point temperature condition (25°C), but varying at low and high points of organic composition in the mobile phase and reaching minimum at center point conditions. An increase in flow rate led to a decrease in theoretical plates, while the opposite trend was observed for temperature. Additionally, slight variations in retention time occurred with changes in temperature, but a significant decrease in temperature resulted in an increase in theoretical plates.

### Optimized chromatographic conditions

**Fig. 4:**
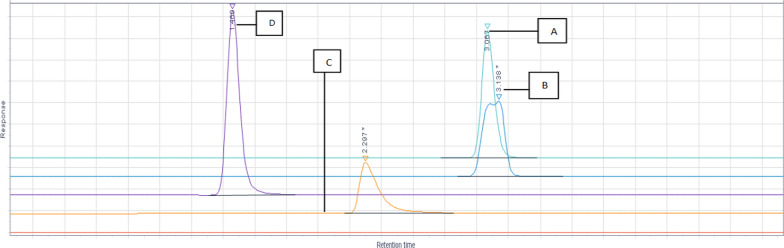
Different Column trials. **A** Phenomenex Luna C 18 250 × 4.6 mm (10 µm). **B** Phenomenex Luna C18 250 × 4.60 mm (5µm). **C** Eclipse plus C18 100 × 4.60 mm (3.5µm). **D** ACE Excel C18 100 × 2.1 mm (2µm)

**Fig. 5:**
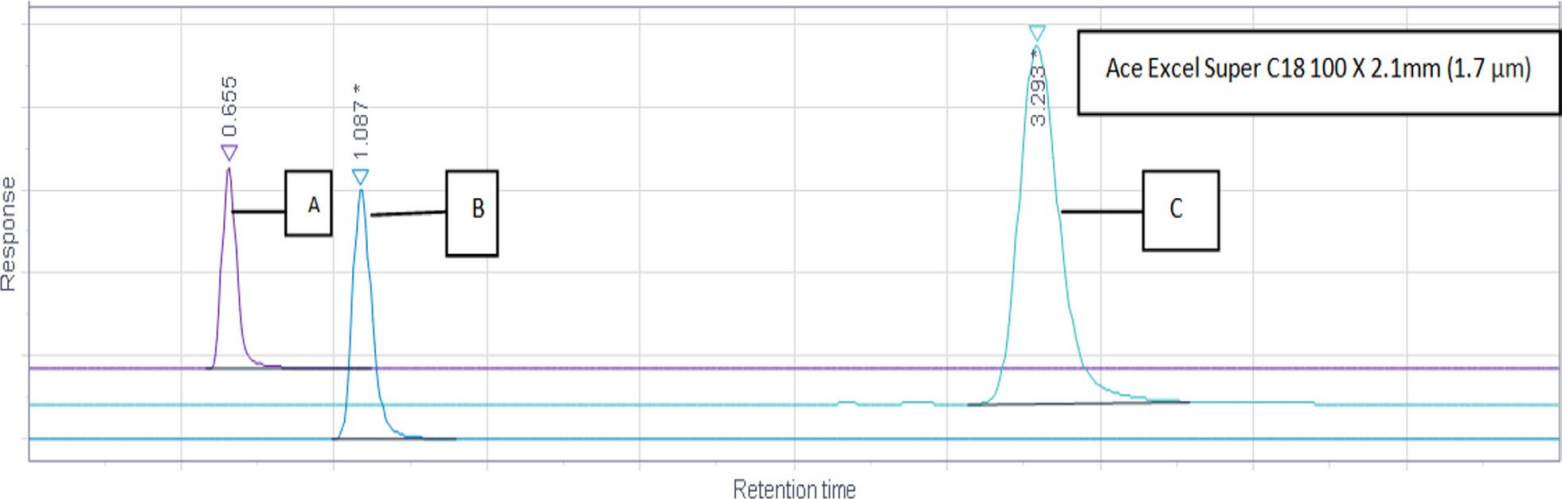
Different mobile phase and flow rate trials. A Mobile phase ACN: MeOH (95:5) and flow rate 0.5 mL/min. B Mobile phase ACN: MeOH (95:5) and Flow rate 0.3 mL/min. C Mobile phase ACN: MeOH (95:5) and flow rate 0.1mL/min

**Fig. 6:**
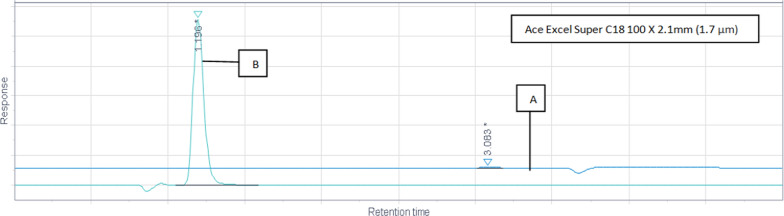
Different mobile phase and flow rate trials. **A** Mobile phase 0.1%TFA in ACN: MeOH (95:5) and flow rate 0.3 mL/min. **B** Mobile phase 0.1%TFA in ACN: MeOH (95:5) and flow rate 0.1 mL/min

**Fig. 7:**
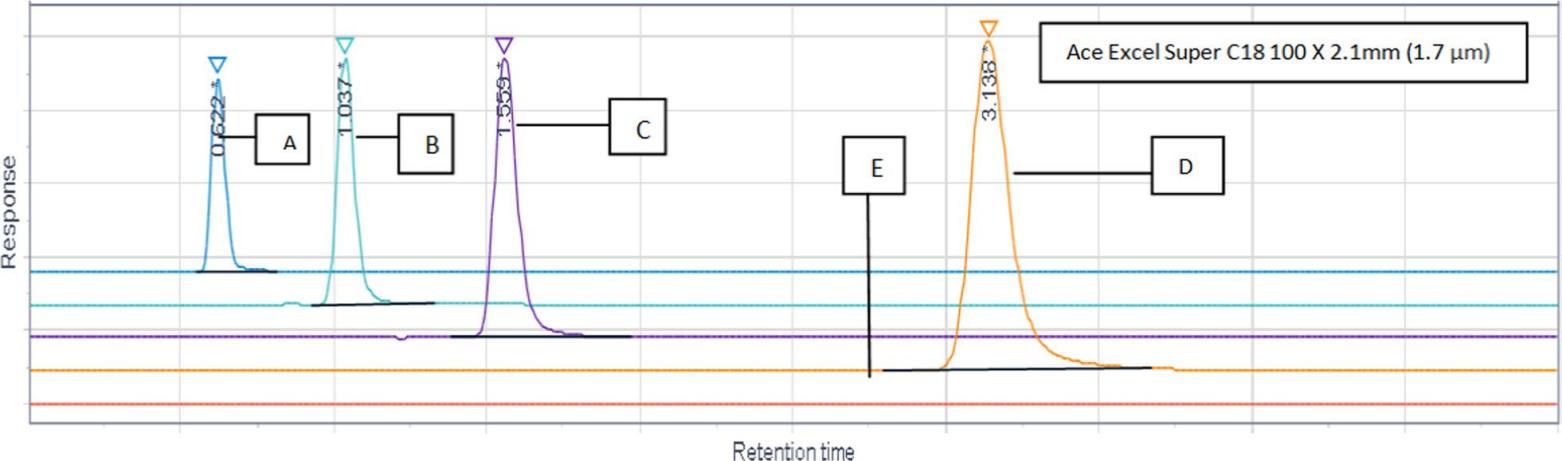
Different flow rate and temperature trials at 45 °C. **A** Flow rate 0.5 mL/min and Temperature 45 °C. **B** Flow rate 0.5mL/min and temperature 45 °C. **C** Flow rate 0.5mL/min and temperature 45 °C. **D** Flow rate 0.5 mL/min and Temperature 45 °C. **E** Blank flow rate 0.5 mL/min and temperature 45 °C

**Fig. 8:**
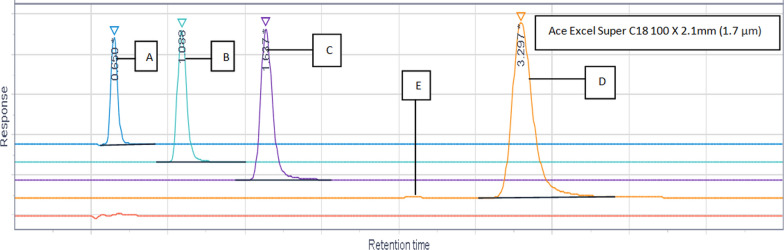
Different flow rate and temperature Trials at 25 °C. **A** Flow rate 0.5 mL/min and Temperature 25 °C. **B** Flow rate 0.5 mL/min and Temperature 25 °C. **C** Flow rate 0.5 mL/min and Temperature 25 °C. **D** Flow rate 0.5 mL/min and Temperature 25 °C. **E** Blank flow rate 0.5 mL/min and Temperature 25 °C

**Fig. 9:**
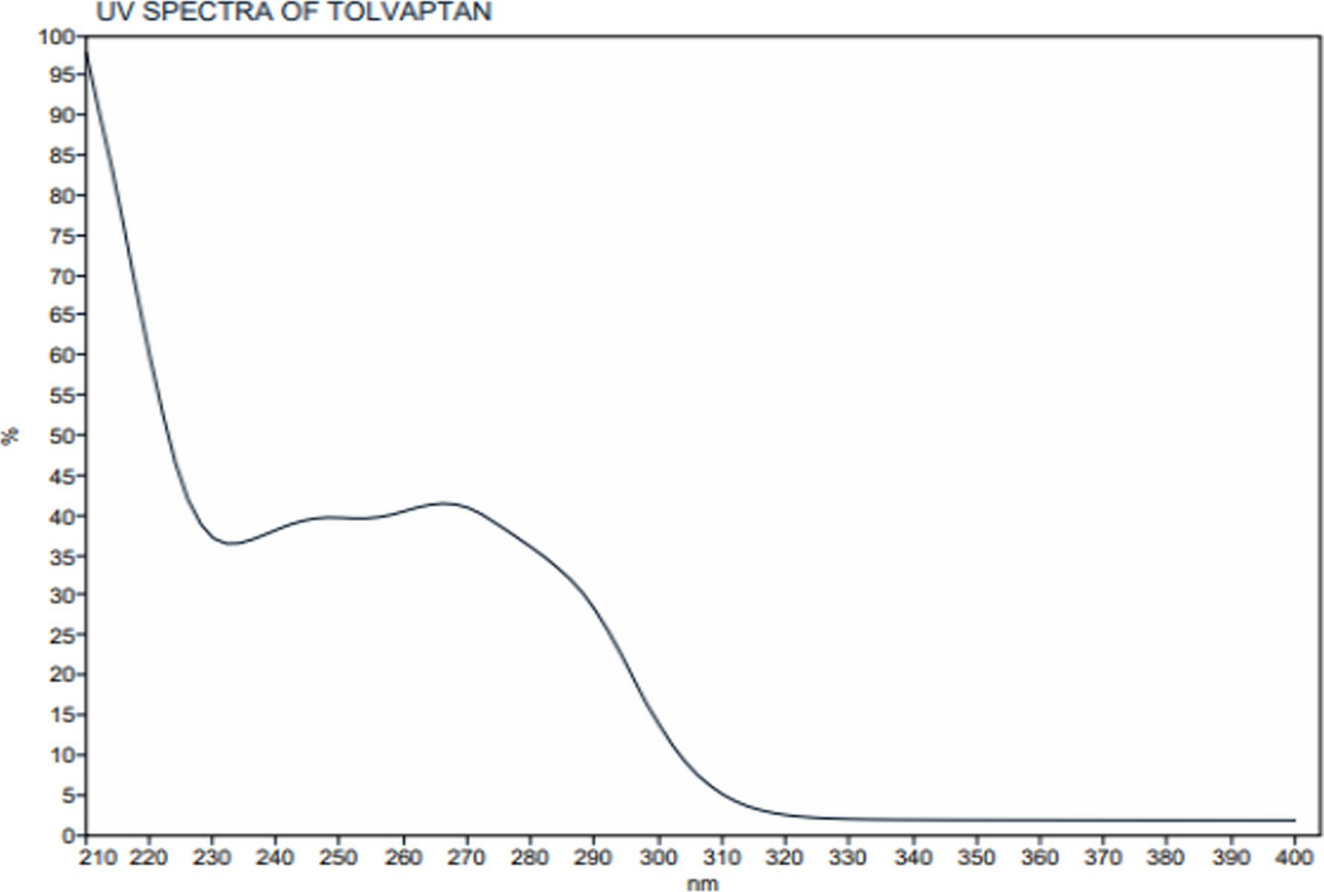
UV spectra of Tolvaptan

Different Column trials were reported in Fig. 4, various mobile phases and flow rate trials were shown in Figs. 5, 6, different flow rate and temperature trials were shown in Figs. 7, 8 and the optimized conditions involved utilizing the ACE Excel Super C18 column (100 mm, 1.7 µm, 2.1 mm) as the stationary phase. A detection wavelength of 220 nm was chosen for the UV–Visible detector because at this wavelength, there was the highest absorption as shown in Fig. 9. The isocratic mobile phase comprised acetonitrile: Water [95:5], passed through the column at a consistent flow rate of 0.2 mL/min at 25 °C with an injection volume of 3 µL for 5 min as total run time.

### Method validation.

**Fig. 10:**
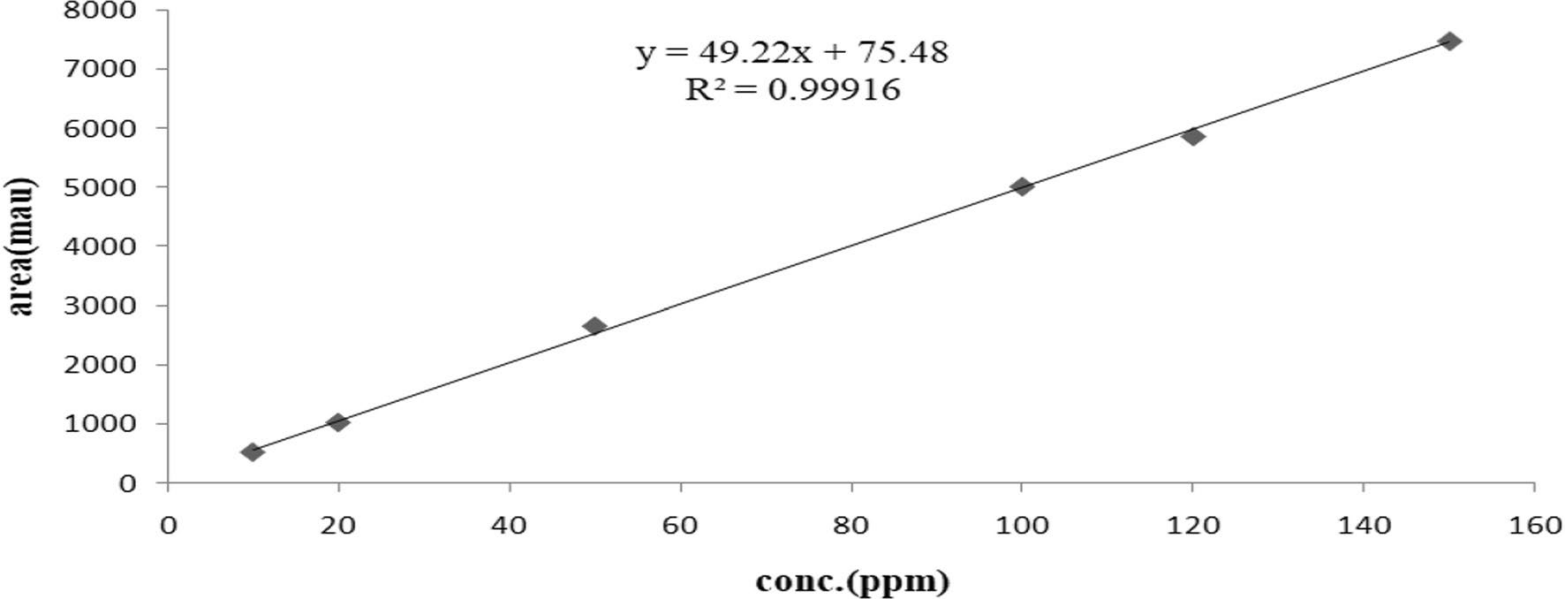
Calibration curve

Linearity: Linearity was confirmed by construction of calibration curves. The calibration curve was displayed in Fig. 10, R2 for tolvaptan within the concentration range exceeded 0.99. Linearity results were shown in Table 5.

**Table 5:** Validation results of proposed UHPLC method for the estimation of Tolvaptan

Parameters	TLP	Acceptable criteria
*Linearity*		0.999
Concentration range (µg/mL)	10–150 µg/mL	
Slope	49.22	
Intercept	75.48	
Correlation coefficient (r)	0.99916	
*Accuracy (% recovery)*		98–102% ± 2.0%
50% mean ± RSD	99.964 ± 0.397472	
100% mean ± RSD	99.775 ± 0.590492	
150% mean ± RSD	100.0793 ± 0.822959	
*Precision*	*RSD (%)*	NMT 2.0%
Method precision	0.4681	
System precision	0.0482	
*LoD*	0.00606 mg/mL	
*LoQ*	0.0183677 mg/mL	
*Robustness*	*RSD (%)*	NMT 2.0%
Flow 0.1 (mL/min)	0.0251	
Flow 0.3 (mL/min)	0.4941	
Mobile Phase_ACN:H_2_O (94:6)	0.1072	
Mobile Phase_ACN:H_2_O (96:4)	0.0819	
Temperature 30 °C	0.0461	
Temperature 20°C	0.2719	
*Solution stability (5 days)*	0.31	NMT 2.0%

LOD and LOQ: The calculation determination of LOD and LOQ from the calibration curve was successfully achieved, and the results were presented in Table 5.

Accuracy: The recovery percentage was calculated for each triplicate and % RSD was calculated, yielding values consistently below 2%. The results for accuracy were given in the Table 5.

Precision: Different type of precision were calculated at different concentrations and the %RSD was calculated which was consistently falling below the acceptable threshold of 2% as outlined in the guidelines provided in Table 5.

**Fig. 11:**
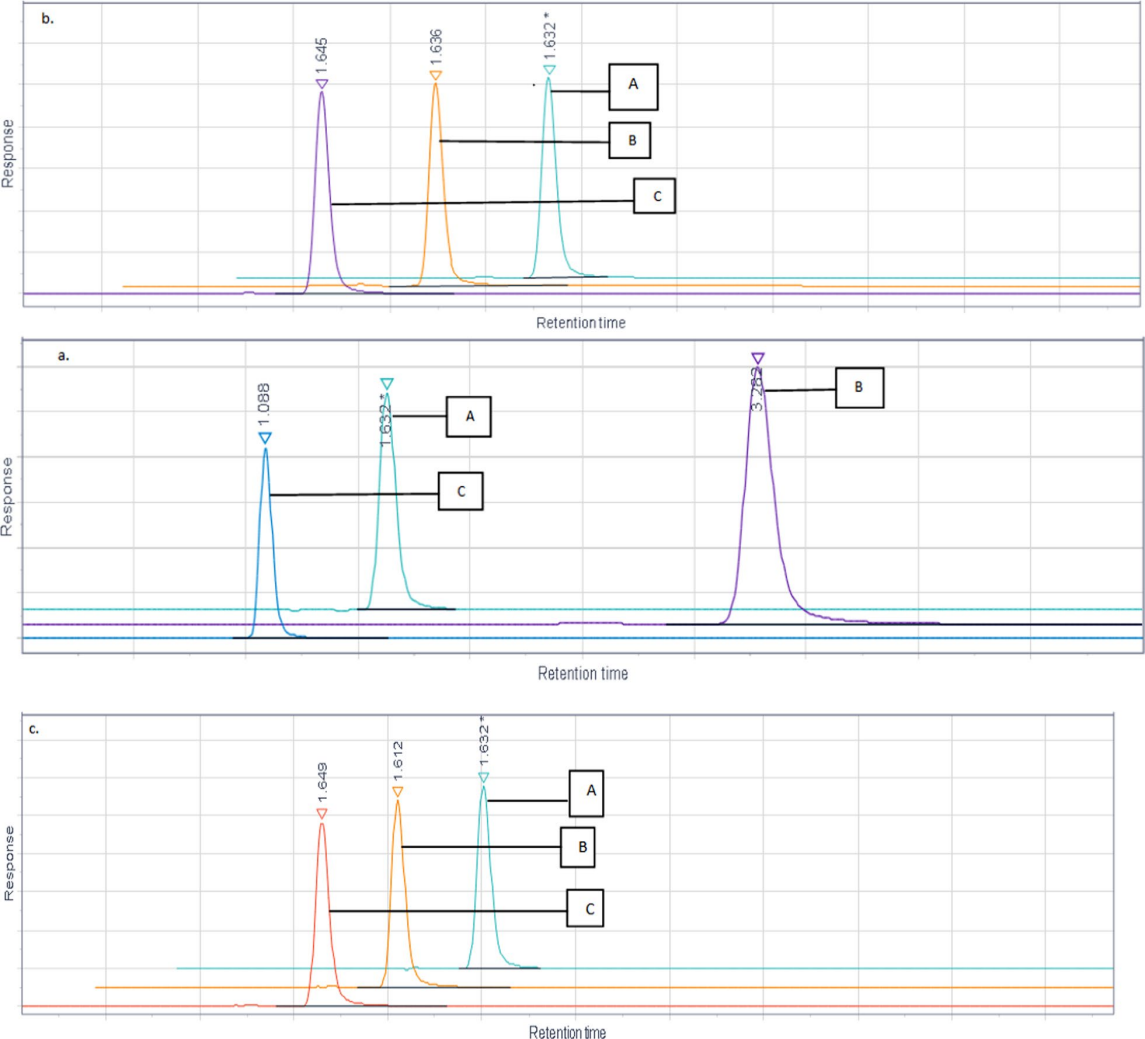
**a** Robustness flow rate. (**A**) Flow rate 0.2 mL/min (**B**) Flow rate 0.1 mL/min. (**C**) Flow rate 0.3 mL/min. **b** Robustness mobile phase. (A) Mobile phase ACN: H_2_O (95:5 v/v) (B) Mobile phase ACN: H_2_O (94:6 v/v) (C) Mobile phase ACN: H_2_O (96:4 v/v). **c** Robustness temperature. (A) Temperature 25 °C (**B**) Temperature 30 °C. **C** Temperature 20 °C

Robustness: Different conditions of robustness were evaluated by making small changes from the original condition, and the resulting %RSD values were presented in Table 5. Chromatograms illustrating these conditions were depicted in Fig. 11.

Solution stability: The percentage change in area was calculated indicating he values within the acceptable range of not more than 2% and no degradation peak was detected as shown in Table 5

**Fig. 12:**
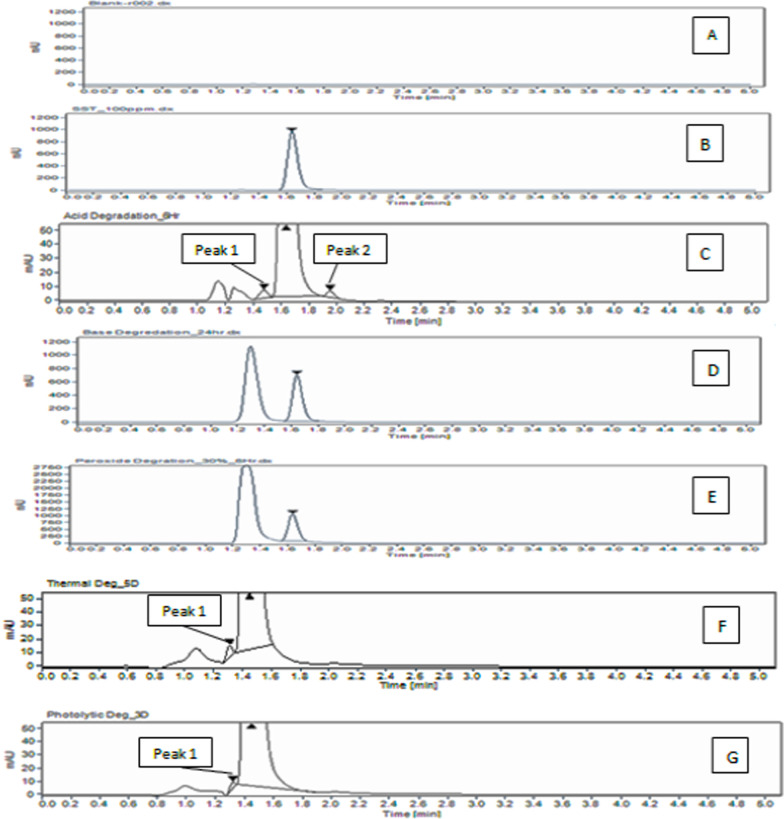
Force degradation study. (A) Blank. (B) Standard. (C) Acid 6 h. (D) Base 24 h. (E) 30% H_2_O_2_. (F) Thermal 5 days. (G) Photolytic 3 days

Force degradation study: The study results of tolvaptan degradation studies were given in the Table 6. Degradation percentage of tolvaptan was noticed in acidic hydrolysis, and excellent separation was attained between the degradants, in acidic condition. Figure 12 shows the force degradation chromatogram.

**Table 6:** Summary of force degradation studies

Name	Time (h)	Assay (%w/w) in degradation sample	Mass balance (assay + total impurity)	Remark	Peak purity
Acid Degradation (0.2 N HCL)	6 h	98.43	99.99	Two degradation peaks peak 1 and peak2 were observed	Pass
Base Degradation (0.2N NaOH)	24 h	99.94	99.98	No degradation was observed	Pass
Oxidation (H_2_O_2_ 30%)	6 h	99.72	100.1	No degradation was observed	Pass
Thermal (105 °C)	5 days	99.53	100.1	One degradation peak 1 was observed	Pass
Photolysis (sunlight)	3 days	99.82	100.3	One degradation peak 1 was observed	Pass

## Method greenness assessment

**Fig. 13:**
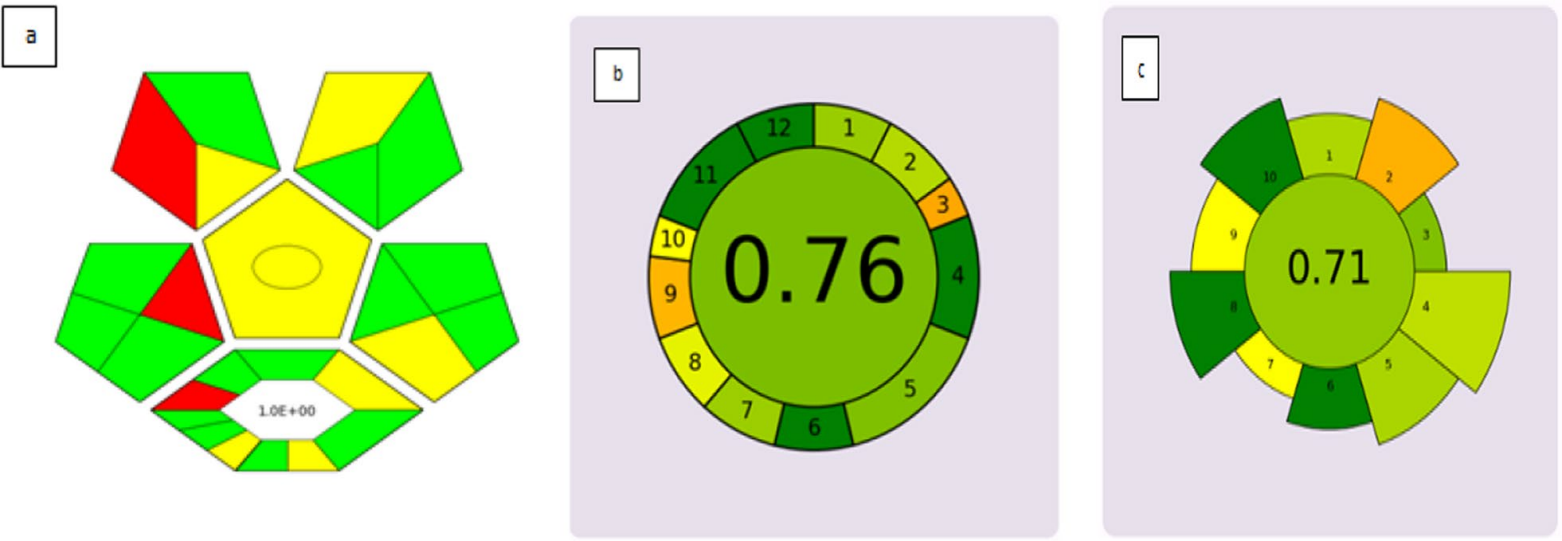
Green assessment tools. **a** Complex Gapi. **b** AGREE tool. **c** AGREE prep

The proposed methodology underwent a comprehensive evaluation using AGREE prep and AGREE, two greenness assessment tools. The outcome of the assessments were analyzed by inputting the procedure followed during the experiments into the software, and the results were visually displayed in Fig. 13. Additionally, we evaluated the suggested approach using the complex GAPI, which is a tool that assigns a score to a technique based on its adherence to certain criteria. The score was represented by a pentagram in a pictogram, with colors of the pentagrams indicating the degree of adherence to specific criteria. Predominantly green and yellow pentagrams in the pictogram suggested that the suggested approach is eco-friendly. Furthermore, we used the EAS score, a comprehensive assessment tool that considers six parameters. After applying penalties as per the tool's guidelines, our technique scored an impressive 75 points. Analytical eco scale penalty points were summarized in Table 7 [11]. In summary, the suggested methodology is both rapid and environmentally friendly, as evidenced by its high scores on complex GAPI, EAS AGREE and AGREE prep tools.

**Table 7:** Analytical eco-scale penalty points

Principle/reagents/chemicals	Penalty points
Water	0
Acetonitrile	12
UHPLC	1
Occupational hazard	3
Sample storage (RT)	0
Sonicator	1
Waste	5
Waste management	3
Sum of total penalty points	25
Analytical eco-scale	75

## Conclusion

A simple and rapid QbD assisted green stability-indicating chromatographic UHPLC method was developed for Tolvaptan. Leveraging AQbD and the Central Composite Experimental Design lead to a notable enhancement in the performance and robustness of the method, ensuring successful separation and estimation of Tolvaptan. The number of experimental runs were reduced by adopting Design-Expert software and validation was carried out in accordance with guidelines of ICH Q2 R (1). The validation of the method covered all parameters, and their adherence to acceptable ranges confirmed the linearity, precision, robustness, and sensitivity of the proposed method results. In the pursuit of a sustainable approach, the AES, Complex GAPI, AGREE tool and AGREE were employed for the green assessment, revealing that the presented method's greenness profile stands out as exceptionally superior. This underscores the method's commitment to environmentally conscious practices, making it a compelling choice for estimation of Tolvaptan.

## Data Availability

The datasets used and/or analysed during the current study are available from the corresponding author on reasonable request.
